# Big data show idiosyncratic patterns and rates of geomorphic river mobility

**DOI:** 10.1038/s41467-025-58427-9

**Published:** 2025-04-05

**Authors:** Richard J. Boothroyd, Richard D. Williams, Trevor B. Hoey, Gary J. Brierley, Pamela L. M. Tolentino, Esmael L. Guardian, Juan C. M. O. Reyes, Cathrine J. Sabillo, Laura Quick, John E. G. Perez, Carlos P. C. David

**Affiliations:** 1 School of Geographical and Earth Sciences, University of Glasgow, Glasgow, UK; 2 Department of Geography and Planning, School of Environmental Sciences, University of Liverpool, Liverpool, UK; 3 Department of Civil and Environmental Engineering, Brunel University London, Uxbridge, UK; 4 School of Environment, University of Auckland, Auckland, New Zealand; 5 National Institute of Geological Sciences, University of the Philippines, Diliman, Philippines; 6 Department of Geography and Regional Research, University of Vienna, Vienna, Austria

**Keywords:** Hydrology, Geography

## Abstract

Big data present unprecedented opportunities to test long-standing theories regarding patterns and rates of geomorphic river adjustments. Here, we use locational probabilities derived from Landsat imagery (1988-2019) to quantify the dynamics of 600 km^2^ of riverbed in 10 Philippine catchments. Analysis of lateral adjustments reveals spatially non-uniform variability in along-valley patterns of geomorphic river mobility, with zones of relative stability interspersed with zones of relative instability. Hotspots of mobility vary in magnitude, size and location between catchments. We could not identify monotonic relationships between local factors (active channel width, valley floor width and confinement ratio) and mobility. No relation between the channel pattern type and rates of adjustment was evident. We contend that satellite-derived locational probabilities provide a spatially continuous dynamic metric that can help unravel and contextualise forms and rates of geomorphic river adjustment, thereby helping to derive insights into idiosyncrasies of river behaviour in dynamic landscapes.

## Abstract (web summary)

In this study, satellite-derived locational probabilities are analysed to unravel records of river adjustment in the Philippines. The data show spatially non-uniform variability in along-valley patterns of geomorphic river mobility.

## Introduction

Systematic appraisals of geomorphic river adjustment attained through digital representations of rivers^1–4^ present enormous potential to test key understandings of river morphodynamics. Fluvial geomorphologists have developed a range of predictive tools to appraise forms and rates of adjustment for different channel pattern types (e.g., meandering and braided^5^, wandering^6^ and anastomosing^7^). Satellite-derived global-scale analyses now highlight hotspots of river extent change (erosion and deposition) when observing the wetted parts of rivers^8,9^. To date, however, few studies have rigorously documented both system- and local-scale geomorphic mobility across the entire active width of river systems^10^, despite the importance of looking beyond the surface waters to the wider riverscape^11–13^.

The locational probability approach pioneered by Graf^14,15^ measures the proportion of time that a channel occupies a particular location. Dependence upon manually digitised georeferenced images restricted initial applications of this approach^16,17^. In the era of big data^18^, digital representations of rivers derived from multi-decade satellite imagery archives provide new opportunities to test predictions in fluvial geomorphology^19–21^. Cloud-based computing platforms such as Google Earth Engine (GEE)^22^ facilitate new ways of working with multi-temporal satellite imagery that can be used to differentiate between riverscape features, including surface water, alluvial sediment and vegetation^1^. Together, these digital possibilities make feasible the systematic appraisal of geomorphic river mobility, realising the potential of Graf’s innovation.

Several factors affect the morphologic evolution of alluvial river channels^23^. At the landscape scale, confinement from adjacent topography interacts with water and sediment inputs to fundamentally control river character and behaviour, restricting the extent to which rivers can laterally adjust^24^. Longitudinal variations in valley width have consequences for hydraulic and geomorphic processes, especially where changes in valley morphology are rapid (e.g., constrictions and expansions^25,26^). Channel pattern type is a product of slope, discharge, bed material size and bank strength^27^. Essentially, this manifests as an energy gradient wherein stream power (the product of slope and discharge) acts on available materials of a given size and composition that flow has transported and deposited along a river course. A characteristic downstream transition from braided and wandering gravel-bed rivers (bedload/mixed load systems) through active mixed load systems (active meandering) to passively adjusting suspended load rivers (passive meandering and anastomosing) is generally hypothesised^28–30^. However, channel pattern types are not mutually exclusive and demonstrate a continuum of variability, being more diverse than implied by discrete channel classification schemes^31^. Big data now allow us to test conventional understandings of decadal geomorphic river adjustments (i.e., channel mobility) and to assess where local factors in the context of valley setting contribute to the nature of lateral channel mobility^32^.

Contemporary technical resources and skillsets now present unprecedented opportunities to use remotely-sensed data of sufficient resolution, reliability and timeline to systematically appraise spatial variability of river patterns and rates of river mobility. Importantly, these opportunities extend to parts of the world that historically have not been subject to such analyses and are under-represented in the literature. By definition, this expands the information/knowledge base with which to synthesise understandings of river diversity and behavioural traits. Here we appraise controls on geomorphic river mobility for 10 of the largest river systems in the Philippines. The Philippines’ diverse tectonics, lithology and climate produce catchments and channels with variable hydro-morphological characteristics^33,34^, high weathering rates^35^, high sediment supply^36^ and rapid lateral change^37^. Such conditions are typical of steep tropical landscapes^38,39^. Recurrent high-flow disturbance events in the Philippines create widespread erosion and flood hazards, posing significant risks to people, property and infrastructure^40,41^. The relatively limited extent to which anthropogenic activities have altered flow and sediment regimes offers an opportunity to use the records of river mobility in the Philippines to advance the understanding of dynamic fluvial morphologies.

Here, we use multi-decade satellite data to appraise geomorphic river mobility over 10^1^ – 10^2 ^km river reaches. We use Landsat imagery to maximise study duration, apply established multispectral indices^42,43^ to delineate active channels and calculate locational probabilities over 600 km^2^ of predominately gravel-bed rivers in 10 Philippine catchments. The specific aims of the paper are to: (1) identify system-specific patterns of geomorphic river mobility, (2) assess local factors that control geomorphic river mobility, and (3) contrast adjustment behaviours for different channel pattern types.

## Results

### Satellite-derived locational probabilities

**Fig. 1:**
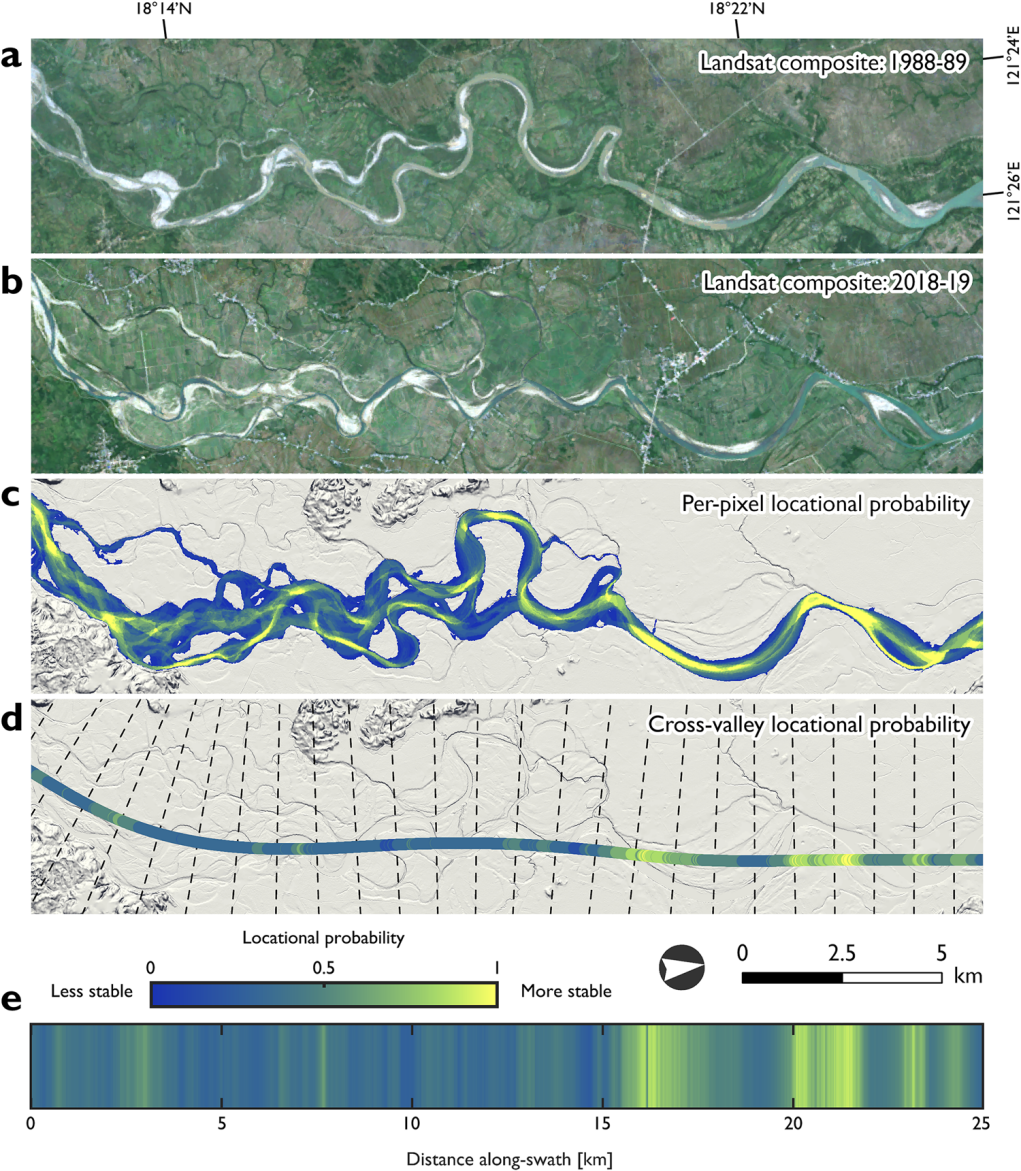
Geomorphic river mobility for a 25 km segment of the Abulug River (Luzon; image centre: 18°18’13.91”N 121°24’30.83”E). Cloud-free satellite imagery at (**a**) the start and (**b**) the end of the analysis period (1988-2019), composited from all available Landsat imagery for two-year time-windows (16 time-windows used for the Abulug River). Geomorphic river mobility expressed as (**c**) the per-pixel locational probability and (**d**) the cross-valley locational probability. Locational probability values closer to 1 indicate that the active channel was consistently positioned at that location through time (i.e., more stable; see Methods section). Cross-valley locational probabilities average the per-pixel location probabilities within a swath in the transverse direction along the valley floor centreline at 0.01 km intervals (dashed lines denote 1 km interval). The same cross-valley locational probabilities are displayed longitudinally in (**e**). Along-valley patterns in geomorphic river mobility are spatially non-uniform; a transition from a less stable (0–15 km) to a more stable channel (15–25 km) is indicated within the example segment. The base maps are (**a**, **b**) composite Landsat imagery and (**c**, **d**) extracts of the hillshaded digital elevation model (see Methods section).

We processed a 32-year record of Landsat satellite imagery between 1988-2019 into two-year time-windows (Fig. 1a, b) and classified active channels as including both wetted channels and unvegetated alluvial deposits; analogous to the bankfull channel extent^44,45^. Having quality checked and manually edited the binary active channel outputs, we calculated per-pixel locational probabilities^14,15^ to indicate the spatial dynamics of geomorphic river mobility (Fig. 1c). Distributions of per-pixel locational probabilities were summarised as cumulative frequency distribution (CFD) curves. Swath profiles represented the average per-pixel locational probability in the transverse direction along a valley floor centreline at regular 0.01 km intervals; we termed these cross-valley locational probabilities (Fig. 1d, e).

### Geomorphic river mobility across spatial scales

**Fig. 2:**
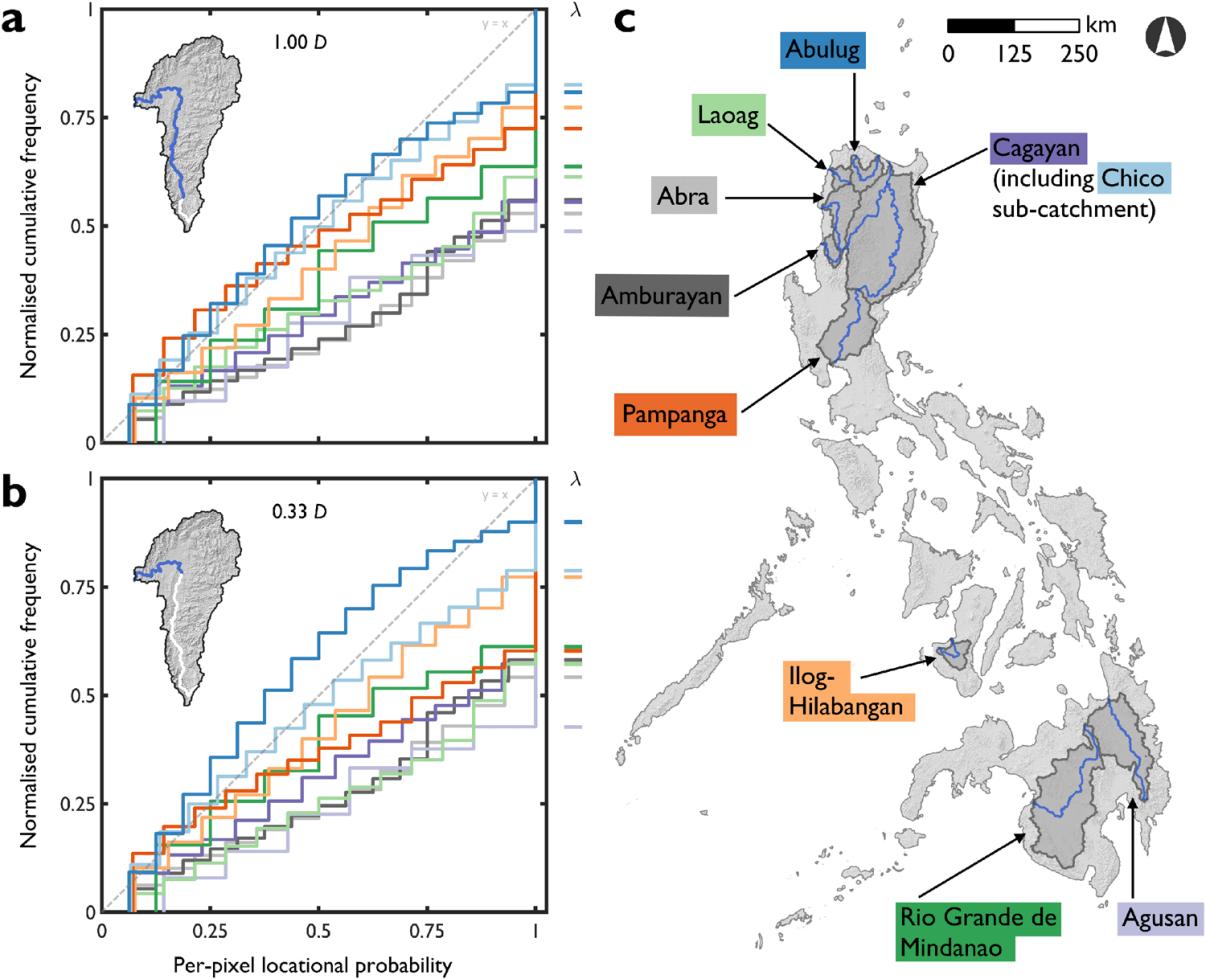
Inter-catchment comparisons of geomorphic river mobility expressed as cumulative frequency distributions (CFD) of per-pixel locational probabilities. **a** Mobility assessed over the entire observable length (1.00 D) of the trunk channel. **b** Mobility assessed over the downstream third of the trunk channel (0.33 D was selected to represent the most complete zone of data, see Fig. 3). Most CFD curves in (**a**) and (**b**) plot below the line of equality, indicating that per-pixel locational probabilities are not evenly distributed against normalised cumulative frequency. Variation in the intercept position (λ) between catchments indicates differences in the proportion of active channel areas that were constantly occupied over the analysis period. **c** Geographic distributions of the trunk channels and catchments used in this study. The colours of catchment labels correspond to the colours of the CFD curves in (**a**) and (**b**). The base maps are (**a**–**c**) the hillshaded digital elevation model (see Methods section).

CFD curves and summary statistics of per-pixel locational probabilities provide first-order representations of river mobility at the system-scale and indicate considerable differences between catchments (Fig. 2 and Supplementary Table 1). For the full observable length of the trunk channel (Fig. 2a), the average per-pixel locational probability ranges from 0.51 (Abulug, least stable) to 0.75 (Abra, most stable). To varying degrees, all trunk channels demonstrate some mobility. The intercept position (λ) where locational probability = 1 denotes the proportion of active channel area constantly occupied over the entire analysis period (for Cagayan, λ = 0.56 and therefore, 44% of the active area is constantly occupied between 1988 and 2019). Approximately half of the active channel areas were constantly occupied for the most stable channels (for example, Abra and Agusan); compared with less than one fifth of the active channel areas for the least stable channels (for example, Chico and Abulug). Although we focus on the full observable length of the trunk channel, the CFD curves are visually similar when the analysis is limited to only the downstream third of the trunk channel (0.33 *D*; Fig. 2b). Only for the Abulug does the shape of the CFD curve indicate relatively less stable behaviour over 0.33 *D* (explained by the highly mobile reaches shown in Fig. 1).

**Fig. 3:**
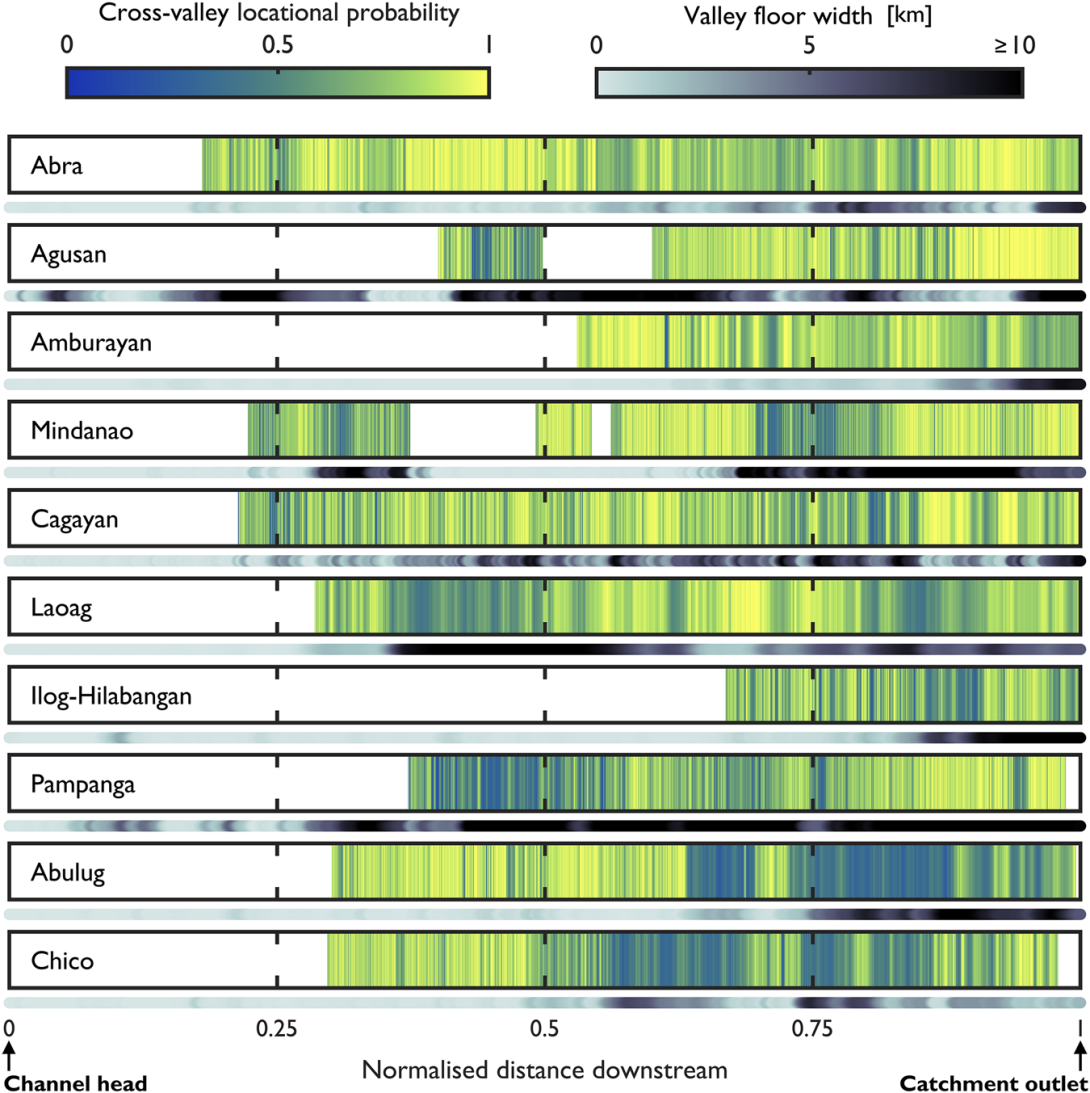
Along-valley patterns in cross-valley locational probability. Individual bars represent regular 0.01 km intervals in the downstream direction. White spaces indicate zones of no data (for example, where the active channel was too narrow to be resolved, see Methods section). Grayscale shading beneath barcode plots represents the valley floor width smoothed over 1 km of river segments using a moving mean. This was mapped from a nationwide IfSAR-derived DEM acquired in 2013 with a 5 m spatial resolution. Trunk channels are sorted by the mean cross-valley locational probability (most stable, top, to least stable, bottom). Visually, some of the prominent hotspots of geomorphic river mobility appear to coincide with topographic settings where the valley floor widens (for example, Laoag, Abulug and Chico). The satellite-derived observations indicate the spatially heterogeneous nature of geomorphic river mobility.

Cross-valley locational probabilities reveal the spatially heterogeneous nature of geomorphic river mobility (Fig. 3). The normalised distance along the valley floor centreline (d) is expressed over the total distance of the valley floor centreline (D). In general, the along-valley patterns are spatially non-uniform; channels are characterised by zones of relative stability interspersed by zones of relative instability, similar to sedimentation zones, and transfer reaches as defined by refs. ^6, 46^. These spatial patterns are exemplified over the ~ 185 km length of the Mindanao channel, whereby discrete zones of instability (0.2-0.375 and 0.7-0.8 d/D) are located between zones of stability. Variance in cross-valley locational probability is high for all trunk channels, ranging from 0.12 (Abra) to 0.23 (Abulug).

Even for the most stable channels (for example, Agusan), discrete pockets of mobility are observed (0.4-0.45 d/D). Hotspots of geomorphic river mobility vary in magnitude, size and location between the 10 channels. Zones of mobility tend to be larger and of higher magnitude for the least stable channels (for example, Pampanga, Chico and Abulug). For Abulug, the zone of instability is located towards the downstream end of the trunk channel (0.7–0.9 d/D). For Pampanga (0.4–0.6 d/D) and Chico (0.6–0.8 d/D), the zones of instability are located further upstream. Results demonstrate the system-specific patterns of geomorphic river mobility.

To consider temporal variability in the spatial patterns, the 32-year record was split into two non-overlapping 16-year time spans (TS1: 1988-2003 and TS2: 2004-2019). Similarity in CFD curves and average per-pixel locational probabilities (µ) indicate stationarity in geomorphic river mobility at the system-scale (Supplementary Fig. 1). Amburayan (µ = 0.85 in TS1, 0.78 in TS2) and Abra (µ = 0.83 in TS1, 0.77 in TS2) became less stable whereas Ilog-Hilabangan became more stable (µ = 0.72 in TS1, 0.77 in TS2). However, the magnitudes of these changes are insufficient to indicate a behavioural shift. To assess the persistence of the spatial patterns, cross-valley locational probabilities were subdivided into non-overlapping 1 km reaches and differences in the locational probabilities between the time spans were calculated. Although many of the reaches are characterised by small differences (i.e., values closer to zero), a substantial proportion of reaches show larger positive or negative differences (> ± 0.2) that indicate a lack of persistence, reflecting temporal variability in geomorphic river mobility (Supplementary Fig. 2). The results show spatially inconsistent patterns of persistence; many reaches are characterised by temporal variability in geomorphic river mobility.

### Local factors as controls on geomorphic river mobility

**Fig. 4:**
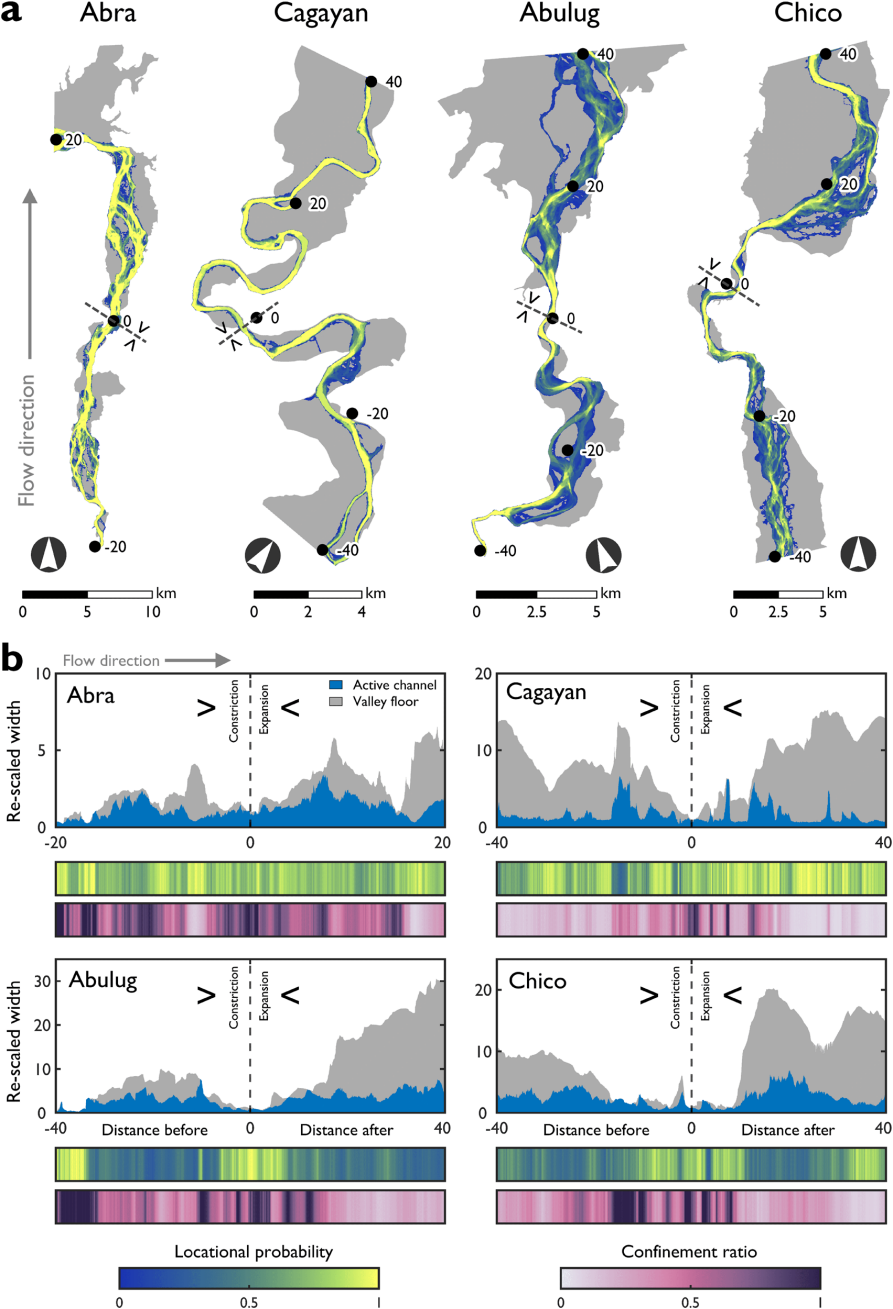
Interplay of local factors (valley floor width, active channel width and confinement ratio) on geomorphic river mobility for four segments with a mid-segment topographic constriction. **a** Geomorphic river mobility expressed as the per-pixel locational probability through the sequence of constriction to expansion; flow direction is from bottom to top. Grey areas show the mapped valley floor. Numbers denote the longitudinal distances before and after the inflection of the constriction/expansion. Coordinate locations at zero longitudinal distances: Abra (17°30'8“N 120°42'59“E), Cagayan (16°32'52“N 121°41'21“E), Abulug (18°9'45“N 121°21'38“E) and Chico (17°39'24“N 121°25'22“E). **b** Local factors in the context of valley setting that contribute to the nature of lateral channel mobility. Longitudinal distances and valley floor widths are re-scaled by dividing by the valley floor width at the inflection (denotated by dashed line). Active channel widths are re-scaled by dividing by the active channel width at the inflection. Locational probabilities use the same colour scale in (**a**) and (**b**). The base maps are (**a**) extracts of the mapped valley floors from the digital elevation model (see Methods section).

Cross-valley locational probability trends can be used to investigate hypotheses of local controls on lateral channel mobility. Segments where the valley morphology rapidly changes through expansions and constrictions are identified on four different rivers, with each segment displaying distinctive patterns of per-pixel locational probability (Fig. 4a). To draw comparisons between the segments, longitudinal distances, valley floor widths and active channel widths are re-scaled (Fig. 4b and Supplementary Table 2). There is marked longitudinal variation in the active channel and valley bottom widths along the segments, with average confinement ratios ranging from 0.25 (Cagayan) to 0.60 (Abra). All segments are characterised by longitudinal variation in geomorphic river mobility, with sites on the Abra and Cagayan more stable (average cross-valley locational probabilities = 0.77 and 0.78) than the Chico and Abulug (0.57 and 0.52).

Spearman correlation coefficients are calculated to measure the monotonic dependence between the local factors (active channel width, valley floor width and confinement ratio) and cross-valley locational probability (Supplementary Table 3). The correlations between active channel width and cross-valley locational probability are strongest and negative (ranging from − 0.80 to − 0.37, *P* < 0.001). Through the sequence of constriction to expansion, narrower parts of the active channel are more stable and wider parts of the active channel are less stable. The strengths of correlations between valley floor width and cross-valley locational probability are weaker and negative (ranging from − 0.69 to − 0.01, *P* < 0.001 to > 0.5). These weaker correlations are unexpected, given that valley confinement has previously been shown to be a dominant control on channel pattern type^47^ and the capacity for lateral channel adjustment^24^. However, the influence is likely limited when confinement ratios are low, and correlations between active channel width and valley floor width are weak (for example, Cagayan). Overall, the patterns of geomorphic river mobility are partly explained by the interplay of local factors where valley morphology rapidly changes, often as a result of changes in lithology and/or faults and other geological structures.

The same local factors were assessed over the full observable lengths of the 10 trunk channels (Supplementary Table 4 and Supplementary Figs. 3 and 4). Spearman correlation coefficients show that the strength and direction of monotonic associations between the local factors and cross-valley locational probability vary between the trunk channels (Supplementary Table 5). Generally, active channel width has the strongest, negative, correlation with geomorphic river mobility, indicating that the narrower parts of the active channel are relatively more stable than the wider parts. The strongest correlations are for the Chico (−0.81, *P* < 0.001) and Abulug (−0.77, *P* < 0.001), these correlations being consistent with previous findings for the sites at specific valley settings (Fig. 4). However, correlations are weaker for other trunk channels (for example, Agusan = −0.19*, P* < 0.001; Laoag = −0.27, *P* < 0.001). Valley floor width has a weaker, negative, correlation with geomorphic river mobility than active channel width. Some rivers show strong positive correlations between active channel width and valley floor  width (for example, Amburayan = 0.92, *P* < 0.001; Abra = 0.85, *P* < 0.001), but others do not (for example, Pampanga = −0.19, *P* < 0.001; Agusan = 0.04, *P* < 0.001). For many of the trunk channels, stable and less stable parts of the river system are distributed over the full range of valley floor widths (Supplementary Figs. 3 and 4).

**Fig. 5:**
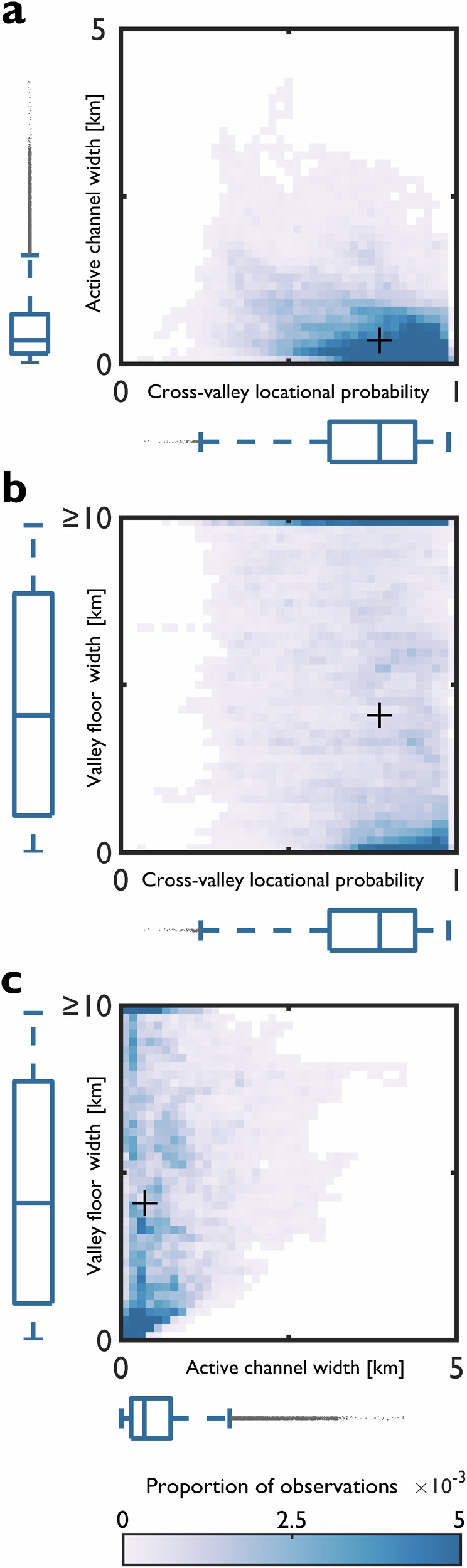
Aggregated observational data from the 10 trunk channels indicate the non-predictable nature of geomorphic river mobility, with considerable noise in the observational record. Relationship between (**a**) active channel width and cross-valley locational probability, (**b**) valley floor width and cross-valley locational probability and (**c**) valley floor width and active channel width. A black marker with a plus sign symbology denotes the median. Marginal boxplots show the median and interquartile range; outliers are defined as values more than 1.5 times the interquartile range.

Control by local factors is further explored by aggregating all of the observational data (Fig. 5). In relation to active channel width (Fig. 5a), a pronounced cluster of more stable rivers (cross-valley locational probability from 0.5 to 1) is found where active channels are narrow (< 1 km). Active channel width appears to be the strongest predictor of geomorphic river mobility, but there is considerable noise in the observational record. The aggregated data (Fig. 5b) also show that there is no particular valley floor width where geomorphic river mobility is accentuated, except for the most confined and narrowest valleys. Geomorphic river mobility, therefore, occurs across a range of valley floor widths. Active channel width is not well predicted by valley floor width (Fig. 5c), and the active channel rarely occupies the entire valley floor (Supplementary Table 4). Across the range of valley floor widths, rivers have the capacity to be more mobile than has been observed. Overall, the aggregated data indicate the non-predictable nature of geomorphic river mobility with considerable noise in the record. Summarising the observational data across all of the scales considered, it is not possible to identify a set of monotonic relationships between local factors and geomorphic river mobility.

### Towards dynamic channel pattern classification

**Fig. 6:**
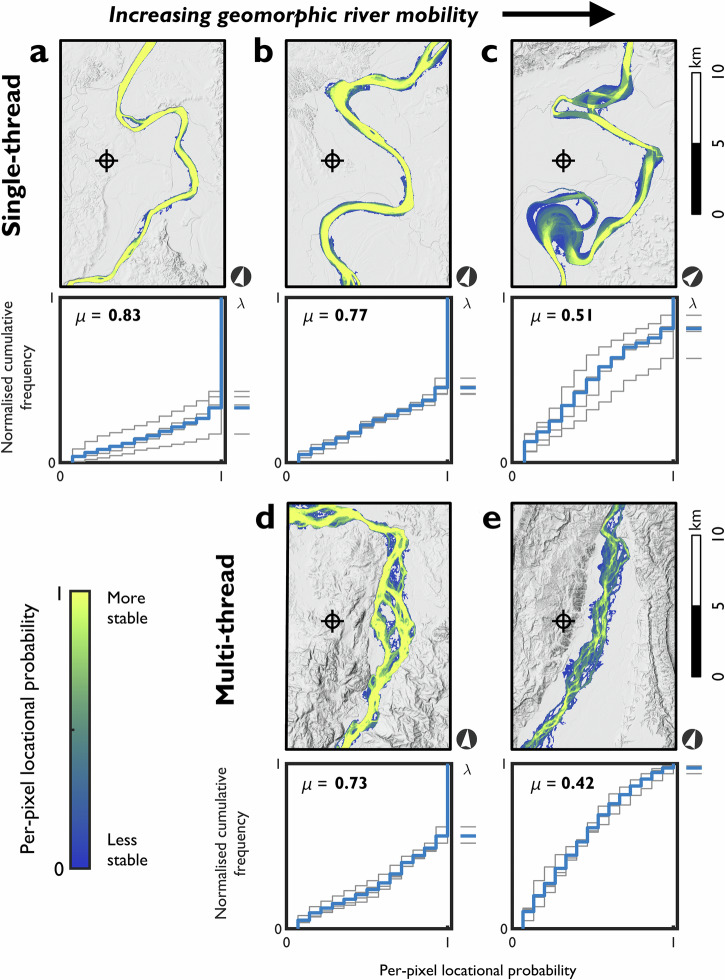
Continuum of geomorphic river mobility behaviours for characteristic segments with single-thread and multi-thread channel patterns. Upper panels show the spatial distribution of per-pixel locational probability across 20 km river segments; lower panels show the corresponding CFD curves. Bold numbers denote the segment averaged per-pixel locational probability (μ). Thick blue lines represent CFD curves for the full segment; thin grey lines represent non-overlapping 5 km reaches. The symbol () denotes coordinate locations close to the selected river segments: (**a**) Cagayan (17°09’52.6”N 121°49’43.9”E); (**b**) Cagayan (17°24’02.2”N 121°44’19.8”E); (**c**) Cagayan (17°49’04.0”N 121°39’31.1”E); (**d**) Abra (17°33’46.7”N 120°41’30.2”E); and, (**e**) Chico (17°32’09.1”N 121°25’50.7”E). For river segments with similar segment averaged per-pixel locational probabilities (for example, **c** and **e**), the shape of the CFD curves and intercept positions (λ) are comparable. We note that inter-reach variability is considerable within segments (for example, **c**), indicating a range of mobility behaviours over relatively short lengths. The base maps are (**a**–**e**) extracts of the hillshaded digital elevation model (see Methods section).

To investigate adjustment behaviour for different channel pattern types, we show five example 20 km river segments that include single-thread and multi-thread channel patterns (Fig. 6), selected for their similar average per-pixel locational probabilities (µ). The examples illustrate the continuum of geomorphic river mobility behaviours seen in the dataset. As the average per-pixel locational probability decreases, shifts in the shape of CFD curves and intercept positions (λ) are observed for all channel patterns. CFD curves tend to be more convex when the segment averaged per-pixel locational probability is higher and more concave when this probability is lower. Both single-thread and multi-thread channel patterns can have similar proportions of the active channel area constantly occupied over the analysis period. For river segments with similar average per-pixel locational probabilities, geomorphic river mobility behaviours are similar irrespective of channel pattern. No relation is found between channel pattern type and rate of adjustment. For example, the single-thread channel (Fig. 6c; μ = 0.51) and multi-thread channel (Fig. 6e; μ = 0.42) have similarly concave CFD curves and comparable intercept positions (λ = 0.19 and 0.03). Therefore, the proportions of the active channel area that are consistently occupied through time appear not to be controlled by channel pattern, as defined using classical static pattern classifications (e.g., refs. ^5–7^). Categorising the channel patterns in these segments using static descriptions would suggest distinct (in this example, meandering and braided) patterns. Our analysis of locational probabilities suggests that the degree of dynamic adjustment over more than three decades is similar for these two rivers. This suggests that the terminology of single-thread meandering and multi-thread braided to describe these rivers needs supplementing with either a metric (μ) to represent their dynamism or an adjective that describes this dynamism relative to the range of possible μ values.

**Fig. 7:**
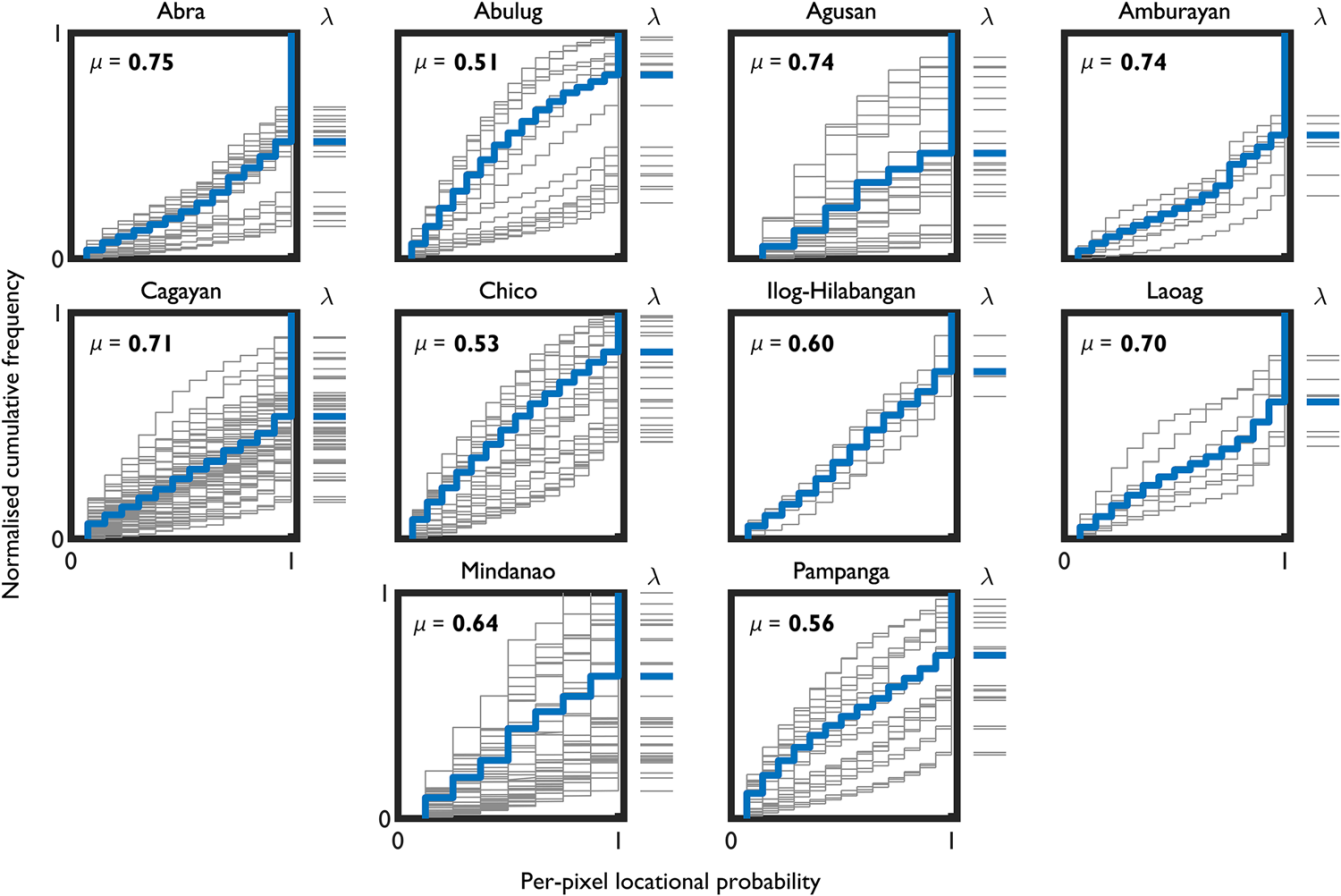
CFD curves over the full observable length of trunk channels (thick blue lines) and over 5 km non-overlapping reaches (thin grey lines). Average per-pixel locational probability (μ) values are shown in bold. The intercept position (λ) indicates the proportion of the active channel area consistently occupied over the analysis period (the proportion is calculated as 1 – λ). The majority of rivers are characterised by large inter-reach differences in CFD curve shape and intercept position, indicating considerable variability in geomorphic river mobility at the system-scale.

Inter-reach variability in geomorphic river mobility is inherent at the system-scale, as shown by differences in the shape of CFD curves and intercept positions between non-overlapping 5 km reaches (Fig. 7). Taking the Cagayan as an example (μ = 0.71), reaches have markedly different CFD curve shapes from convex to concave and intercept positions from 0.11 to 0.84. The CFD curves of individual reaches deviate from the CFD curve for the full observable length of the trunk channel (i.e., different reaches have different mobility behaviours). Even for the systems that are identified to be most stable overall (for example, Abra and Agusan), marked inter-reach differences in mobility exist. Ilog-Hilabangan is the only river with limited inter-reach variability, where all CFD curves have similar shapes and intercept positions (i.e., all reaches have similar mobility behaviours). The system-scale analyses indicate the continuum of geomorphic river mobility behaviours that are observed regardless of channel pattern type, further consolidating our findings (Figs. 3–6) of the idiosyncratic nature of geomorphic river mobility from the reach- to the system-scale.

## Discussion

Our quantification of geomorphic river mobility behaviour as satellite-derived locational probabilities provide insights into the spatial variability of lateral adjustment, offering an important interpretative tool for further investigation of specific river dynamics. In assessing dynamism across the whole active width of rivers, including the wetted channel and unvegetated alluvial deposits, our results show spatially non-uniform variability in along-valley patterns of geomorphic river mobility, with zones of relative stability interspersed with zones of relative instability (Fig. 3). Patterns and rates of adjustment vary within a given reach and across different reaches, pointing to the idiosyncratic nature of geomorphic river mobility across spatial scales^48^. These idiosyncrasies have implications for the practice or conduct of geomorphic enquiry, highlighting the complementary and dual importance of timeless (process-based, linear, morphodynamical) and timebound (historical, contingent, emergent, complex, non-linear) geomorphology^48–50^.

We find it is not possible to identify a set of monotonic relationships between either active channel width or valley floor width and geomorphic river mobility. These factors are locally important where the valley morphology rapidly changes (Fig. 4), but when viewed at the system-scale, these associations are weakened (Fig. 5). Although local factors are important for lateral adjustment processes, many factors operating over different scales influence geomorphic river mobility^51,52^. Predicting mobility is difficult because of the number of variables involved and the complexity of their interactions in the natural environment^53^. This includes the interplay of hydrological variability, sediment supply and vegetation dynamics. Direct and indirect anthropogenic activities further add to the challenge^54^. Complex landscape systems produce outcomes that may be unpredictable at particular places and times^55^ although with predictable time- and space-integrated statistical properties. Big data provides a growing record that increasingly supports the emergence of geomorphology as a data-rich, predictive science but initial findings of tests of long-standing theories and principles highlight the complexity of responses^56^. Noting the limited set of factors investigated, our findings indicate non-predictable patterns of geomorphic river mobility, with considerable noise in the observational record. The idiosyncratic geomorphology reaffirms the primacy of timebound relations. Although technological advancements enhance our ability to comprehend complex landscape systems^57^, the big data presented does not enable the prediction of lateral adjustments in a “naughty” world^58^.

Satellite-derived locational probabilities provide a way of exploring channel pattern classification that is spatially continuous and explicitly accounts for the observed dynamism. Importantly, the approach outlined here enables systematic (consistent) analysis of adjustment (mobility) for all channel pattern types. We find no relation between channel pattern type and rates of adjustment (Fig. 6). Inferred geomorphic river mobility behaviours may be similar between channels that would ordinarily be categorised as distinct if described using static descriptions, or may differ between channels that would have identical static descriptions. More rigorous quantitative approaches for process-based channel pattern classification that incorporate mobility are needed to interpret spatial and temporal patterns^59,60^. The use of satellite-derived locational probabilities as a spatially continuous dynamic metric (µ) presents opportunities to realise this ambition. This refinement of channel pattern descriptions to include mobility will support the reassessment of existing empirical^61^ and theoretical^62,63^ approaches to explain channel pattern, adding temporal change to future analyses of statistical properties of river planform^64,65^. Moreover, data-driven approaches to the systematic analysis of river mobility have profound implications in determining ‘what to measure against’ in the appraisal of river health and resilience^66,67^.

Such analyses can support appropriately contextualised place-based (catchment-specific) management applications^55^ that work with the river (i.e., Nature-based Solutions), giving space, time, energy and materials for rivers to adjust, erode, flood, function, change and evolve^68–70^. Estimating the mobility space requirements of rivers is challenging, especially for geodiverse rivers with varying hydro-bio-geomorphological properties^71,72^. Our findings highlight the complex spatial patterns of geomorphic river mobility and the difficulties of relating local factors to these observations. Simple predictive approaches for estimating mobility space requirements based on measurable factors such as confinement are unlikely to provide an adequate solution. Rather, satellite-derived locational probabilities transform our ability to visualise and quantitatively delimit the space occupied by the channel over multiple decades, regardless of channel pattern type. However, we note that the locational probability approach implicitly assumes the temporal stationarity of the datasets. We observed system-scale stationarity in geomorphic river mobility but identified spatially inconsistent patterns of persistence. The temporality of planform changes should be further explored as real river dynamics are a product of space-time interactions. Insights from satellite-derived locational probabilities offer a way of unravelling records of river adjustment. The digital representations can be recurrently updated as the temporal duration of the satellite imagery record extends, providing a “living database” to make data-driven river management decisions in dynamic landscapes.

## Methods

### Catchment and trunk channel context

Trunk channels from 10 Philippine catchments were included in the analysis (Supplementary Tables 6 and 7). The geographical coverage included the three main island groupings of Luzon, Visayas and Mindanao. The selected catchments were large (catchment area > 1250 km^2^) with a range of topographic, climatic, geologic and land cover properties. The catchment area varied over two orders of magnitude (from 1262 to 27684 km^2^) and most catchments were characterised by high relief (80% of catchments relief > 2350 m). Catchment average slope varied from 9 to 28° whilst the average channel slope ranged from 0.01 to 0.09 m/m. The catchments have mean annual rainfall totals in the range of 1881 to 2450 mm, but this is variable across individual catchments (maximum range in mean annual rainfall within a catchment = 1578 mm). The catchments are distributed across all four of the major zones of the modified Coronas climate classification^73^, with tropical cyclones more frequently crossing catchments in the northeastern part of the Philippines than the southwestern part^74^. The primary lithology varies between catchments, including sandstone, shales, reef limestone (3 catchments), recent deposits (3 catchments), undifferentiated metavolcanic (2 catchments), marl, reworked tuff, pyroclastic (1 catchment) and Pliocene-Pleistocene limestone (1 catchment). The primary land cover includes annual crops (4 catchments), wooded grassland (3 catchments), shrubs (1 catchment), closed forest (1 catchment) and open forest (1 catchment).

### Extracting binary active channel masks from Landsat satellite imagery

We used Google Earth Engine (GEE) to extract binary active channel masks from Landsat satellite imagery. The active channel is analogous to the bankfull extent^44,45^ and includes the wetted channel and unvegetated alluvial deposits. Vegetated bars and islands are excluded as these may be anywhere on a continuum from being permanent features to bars that experience annual cycles of seasonal vegetation growth and erosion. For the 10 catchments we defined the trunk channel (see topographic analysis section), applied a buffer with a 3 km radius to encompass the active channel and imported the shapefiles into GEE. Using the shapefiles as the regions of interest (ROI) for analysis, we selected all available atmospherically corrected Landsat 5, 7 and 8 surface reflectance imagery for two-year time-windows between 1988 and 2019 (Supplementary Fig. 5). The two-year time-windows started on 1 January, with a total of 16 time-windows over the analysis period (for example, time-window 1: 01/01/1988 to 31/12/1989; time-window 2: 01/01/1990 to 31/12/1991). A two-year time-window was selected to maximise the opportunity for cloud-free acquisitions, necessary for classifying the active channel from optical satellite imagery. This was because the Philippines represents a challenging environmental setting with highly variable cloud cover conditions^75^. We constructed image collections containing all available Landsat imagery for each time-window and applied the CFmask algorithm to each image based on pixel quality assessment, to mask obstructions from cloud and cloud shadow^76^. Only cloud-free pixels were retained, with bicubic resampling applied on a per-image basis to smooth the representation of the river channel^77^. Cloud-free pixels were retained in all months of the year, covering the full range of hydro-meteorological conditions (Supplementary Fig. 6).

Different multispectral indices support highly differentiated fluvial geomorphology applications^78,79^. We classified the active channel using the normalised difference vegetation index, NDVI^42^, and the modified normalised difference water index, MNDWI^43^. An approach using relational operators between the NDVI and MNDWI has previously performed well in classifying active channels in the vicinity of large river bridges in the Philippines; with the reported classification accuracy greater than 80%^40^. Moreover, an NDVI threshold of 0.2 is established in the literature for identifying dense riparian vegetation^80^. In the current application, we computed pixel-wide NDVI and MNDWI values for all cloud-masked images in the time-window collections, thresholded and selected pixels where (i) MNDWI ≥ − 0.4 and (ii) NDVI ≤ 0.2, rescaled the selected pixels to a value range between [0,1] and reduced the image collection to a single image based on the 10^th^ percentile of values. The percentile reducer was chosen as a consistent approach for reducing the time-window image collections that contained a variable number of images to a single output image. The 10^th^ percentile was selected following sensitivity testing; it provided the most realistic representation of active channel extents across the 10 catchments. In the final GEE processing step, the single output images for NDVI and MNDWI in each time-window were converted into binary masks (presence/absence form) with the Boolean (‘and’) operator applied to generate the intersection of the masks. The resulting binary active channel masks were exported to Google Drive as GeoTIFF files.

### Cleaning and quality checking the binary active channel masks

The binary active channel masks contained misclassified artefacts that did not belong to the river channel (for example, built-up areas and agricultural fields). We applied standard image processing techniques in MATLAB to remove these artefacts and clean the binary images. First, disconnected areas containing < 500 pixels were assumed to have been erroneously classified and removed. Next, a disk-shaped structuring element with a radius of two pixels performed a single iteration of morphological closing to eliminate small gaps and smooth jagged edge representations^81^. In the final processing step, disconnected areas containing < 500 pixels were removed as these represented large, isolated features positioned away from the trunk channel. The final binary active channel images for each time-window were reviewed in GIS to sense-check the data. A manual editing step was necessary to remove connected locations that did not correspond with the main trunk channel (e.g., adjoining tributary channels). Given the 30 m spatial resolution of the Landsat imagery, we acknowledge that narrow (< 30 m) secondary channels are unlikely to be resolved in the current processing workflow.

Only binary images where the active channel was correctly resolved were included in the analysis of geomorphic river mobility. The amount of satellite imagery in the Landsat archive is not constant from year to year, geographically, or among sensors^82^, meaning that ‘data poor’ time-windows exist (i.e., fewer acquisitions available to delineate the active channel). Coupled with variation in the availability of cloud-free acquisitions at different locations across the Philippines, binary images of sufficient quality were not available at all locations for all time-windows (Supplementary Table 8). Catchments with the fewest usable images were located on the island of Mindanao (Agusan and Mindanao), indicating a geographic control on data availability (for example, greater cloud obscuration or fewer Landsat acquisitions in these locations).

### Quantifying geomorphic river mobility using locational probabilities

We calculated the locational probability of active channels to quantify geomorphic river mobility^14,15^. Multi-temporal sequences of manually digitised georeferenced images (for example, historical maps^16^ and aerial photographs^17,83,84^) have been used to calculate locational probabilities and identify river planform dynamics at reach- to segment-scales (10^−1^-10^2 ^km). We calculated locational probabilities from binary active channel masks derived from multi-temporal Landsat imagery. We calculated the locational probability as:

1 $$p=\left({W}_{1}{F}_{1}\right)+\left({W}_{2}{F}_{2}\right)+\ldots \left({W}_{n}{F}_{n}\right)$$

where $p$ is the final locational probability for each pixel of the active channel, ${F}_{n}$ is the binary active channel occurrence at time-window $n$, equal to 1 when a pixel is occupied by the active channel or 0 when a pixel is unoccupied, and ${W}_{n}$ is the weighting value assigned to time-window $n$. The weighting values were calculated as:

2 $${W}_{n}=\frac{1.00}{x}$$

where $x$ is the total number of time-windows (varies per catchment; Supplementary Table 8). We assigned equal weighting to the time-windows because we assumed that each time-window represents a different morphological condition following a bankfull event (i.e., bankfull flow has occurred within the two-year period). Consequently, each time-window represents the product of an event-driven change, regardless of the time between events.

Locational probabilities $({lp})$ ranged between 0 (never occupied by the active channel) and 1 (always occupied by the active channel). Where locational probabilities are low (0 < ${lp}$ < 0.25), the active channel has infrequently occupied the pixel. Where locational probabilities are high (0.75 < ${lp}$ < 1), the active channel has frequently occupied the pixel. Lower locational probabilities indicate channel mobility, whereas higher locational probabilities indicate channel stability. We resampled the locational probability images to 10 m spatial resolution, to match the resolution of the topographic data.

### Integrating topographic and geomorphic river mobility analyses

We used a nationwide digital elevation model (DEM) acquired in 2013 and generated through airborne Interferometric Synthetic Aperture Radar (IfSAR) for topographic analysis. The DEM has 5 m spatial resolution and 1 m root-mean-square error vertical accuracy^85^. We used TopoToolbox V2 to resample the DEM to 10 m spatial resolution (due to computational processing constraints) and delineated catchments and stream networks with a minimum drainage area of 1 km^2^ using standard flow-routing algorithms^86^. For each of the 10 selected catchments, we calculated fundamental topographic metrics (Supplementary Table 6) and extracted trunk channels (defined as the longest stream within the catchment). The trunk channels were used to define the regions of interest (ROI) to complete the multi-temporal satellite imagery analysis in GEE.

Valley floors were manually mapped in GIS using the nationwide DEM. We defined the valley margins morphologically, by identifying breaks in slope from relatively flat, low elevation areas to relatively steep hillslopes^24,37,87^. The valley floor included active floodplains and terraces, in addition to other valley floor landforms (for example, fans). It was not feasible to distinguish additional margins (for example, anthropogenic or channel margins) at the catchment-scale using the topographic data available.

We used the SWATHobj function in TopoToolbox V2 to sample attribute values for per-pixel locational probabilities along the valley floor centreline. We set the SWATHobj width to 10 km to fully encompass the transverse width of the active channel and set the resampling distance to 0.01 km (‘dx’ parameter) to extract attribute values at along-valley intervals equal to the resampled DEM resolution. Attribute values for per-pixel locational probability were extracted across the transverse width of the swath profile at the regular intervals and then averaged to provide a generalised output (termed the cross-valley locational probability). Active channel width was calculated from the non-zero per-pixel locational probability values across the transverse width of the swath profile at regular intervals. The confinement ratio was calculated as the active channel width divided by the valley floor width. Distances along the swath profile were normalised by the total swath length; this provided a standard framework to compare along-valley patterns of geomorphic river mobility and valley floor width between multiple catchments.

## Supplementary information

Supplementary Information

## Data Availability

The locational probability data generated in this study have been deposited in the NERC Environmental Information Data Centre (EIDC) along with supporting documentation (10.5285/a2bcc66e-4dcc-4ed1-897d-cdf36dde246d).

## Code availability

Google Earth Engine and MATLAB codes for processing the locational probability data have been deposited in the NERC Environmental Information Data Centre (EIDC) along with supporting documentation (10.5285/a2bcc66e-4dcc-4ed1-897d-cdf36dde246d).
