# Supplementary material for: Big data show idiosyncratic patterns and rates of geomorphic river mobility
Source: Nat Commun. 2025 Apr 5;16:3263. doi: 10.1038/s41467-025-58427-9 (PMC11972300; doi:10.1038/s41467-025-58427-9)
Supplement: Supplementary file 1 — Supplementary Information [file 41467_2025_58427_MOESM1_ESM.pdf]

Supplementary Information

Big data show idiosyncratic patterns and rates of geomorphic river mobility

Richard J. Boothroyd[1,2]*, Richard D. Williams[1], Trevor B. Hoey[3], Gary J. Brierley[4], Pamela L.M. Tolentino[1,5], Esmael L. Guardian[5], Juan C.M.O. Reyes[5], Cathrine J. Sabillo[5], Laura Quick[1], John E.G. Perez[5,6] and Carlos P.C. David[5]

[1] School of Geographical and Earth Sciences, University of Glasgow, Glasgow, UK
[2] Department of Geography and Planning, School of Environmental Sciences, University of Liverpool, Liverpool, UK
[3] Department of Civil and Environmental Engineering, Brunel University London, Uxbridge, UK
[4] School of Environment, University of Auckland, Auckland, New Zealand
[5] National Institute of Geological Sciences, University of the Philippines, Diliman, Philippines
[6] Department of Geography and Regional Research, University of Vienna, Vienna, Austria

* Correspondence to richard.boothroyd@glasgow.ac.uk or richard.boothroyd@liverpool.ac.uk

Contents of this file

Supplementary Figures 1-6
Supplementary Tables 1-8

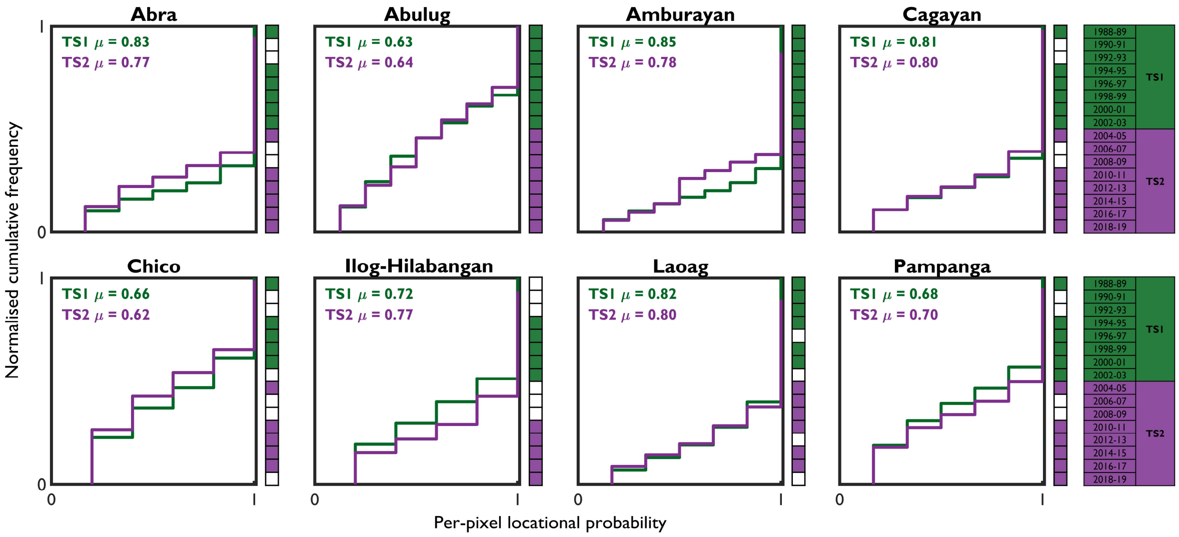

Supplementary Figure 1: CFD curves over the full observable length of eight trunk channels, for two non-overlapping 16-year time spans (TS1: 1988-2003 and TS2: 2004-2019). Average per-pixel locational probability (μ) values are shown in bold. Marginal coloured boxes relate to the time-windows included in each time span. An equal number of time-windows were included in TS1 and TS2 for each trunk channel. Due to the limited temporal coverage of active channel imagery for Agusan and Mindanao (Supplementary Table 8), these channels were not included in the temporal analysis. Similarity of the CFD curves and average per-pixel locational probabilities indicate stationarity in geomorphic river mobility at the system-scale.

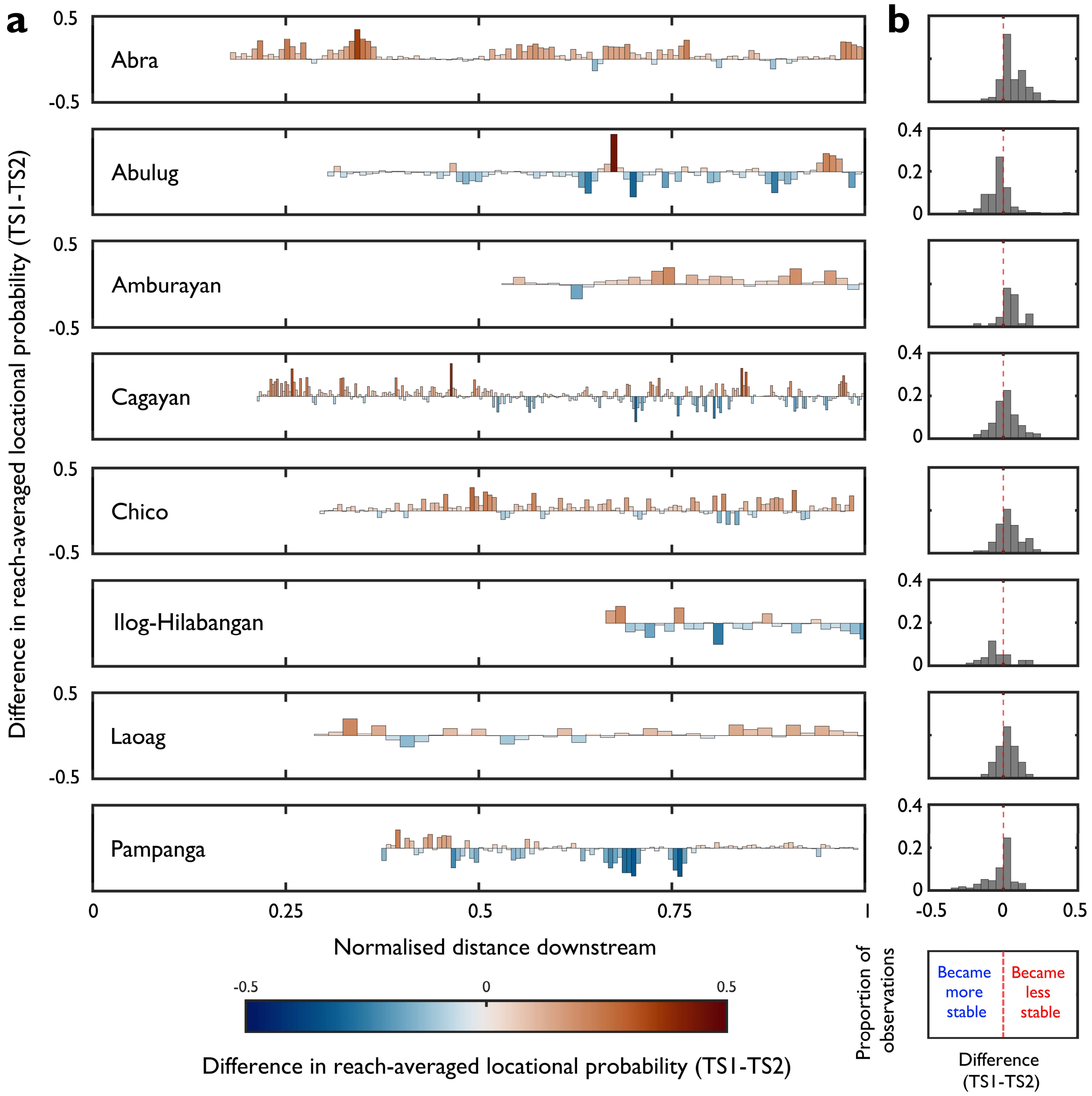

Supplementary Figure 2: Persistence in the spatial patterns of geomorphic river mobility as (a) along-valley differences in reach-averaged locational probability between TS1 and TS2 and (b) distributions of the differences. Cross-valley locational probabilities were subdivided into non-overlapping 1 km reaches and the differences in reach-averaged locational probability were calculated (TS1-TS2). Positive difference values mean the reach became less stable; negative difference values mean the reach became more stable. Although many of the reaches are characterised by small differences, a substantial proportion show larger positive or negative differences that indicate a lack of persistence, reflecting temporal variability in geomorphic river mobility.

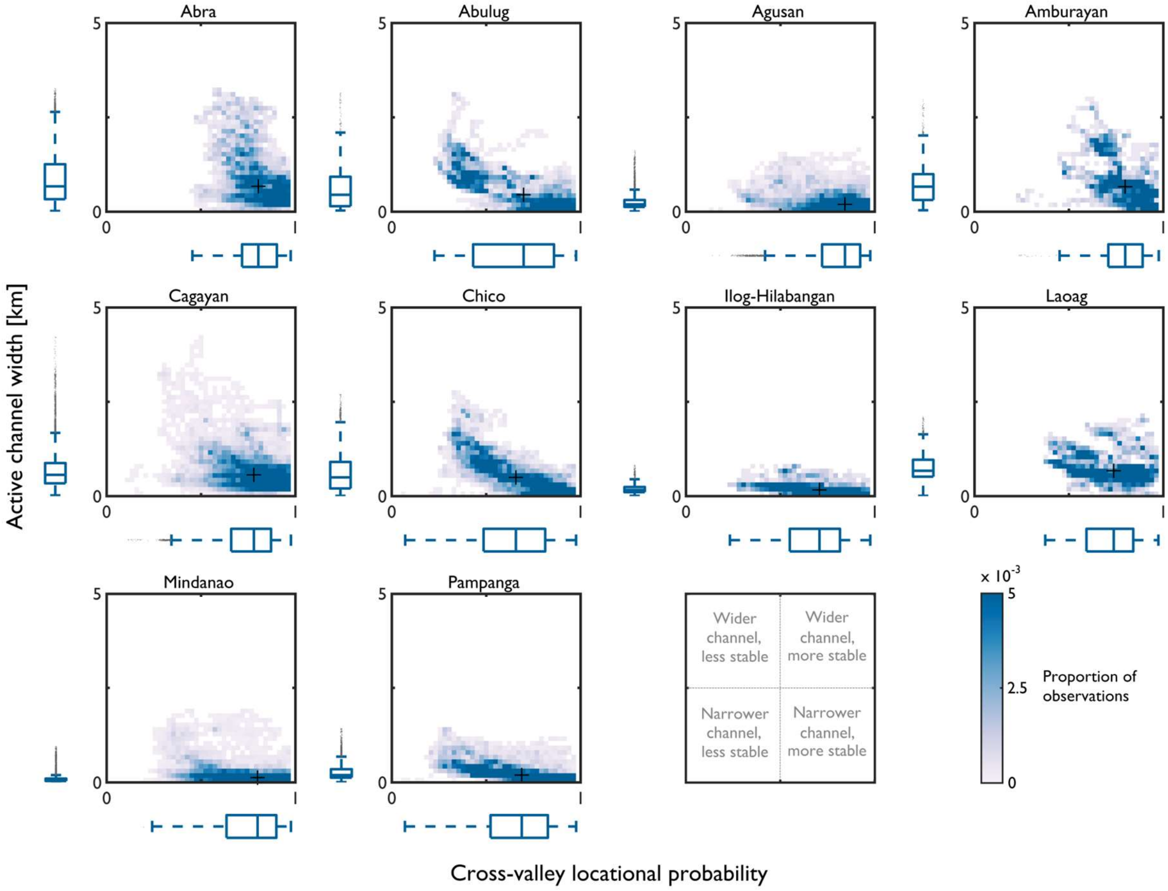

Supplementary Figure 3: Proportion of cross-valley locational probabilities displayed as a function of active channel width. Bin sizes are 0.025 for locational probability and 0.0125 km for active channel width. Marginal boxplots show the median and interquartile range; outliers are defined as values more than 1.5 times the interquartile range. Black marker with plus sign symbology denotes the median. Stable parts of the channel tend to be associated with narrower active channel widths. For one of the least stable channels (Abulug), two discrete peaks in frequency are observed where (i) locational probabilities are lower (< 0.5) and active channel width is greater (> 1 km) and (ii) locational probabilities are higher (> 0.65) and active channel width is lower (< 1 km). For some rivers (for example, Cagayan) there exists considerable noise in the relationship between active channel width and locational probability. For other rivers (for example, Abra) locational probabilities are generally high but distributed over the full range of active channel widths.

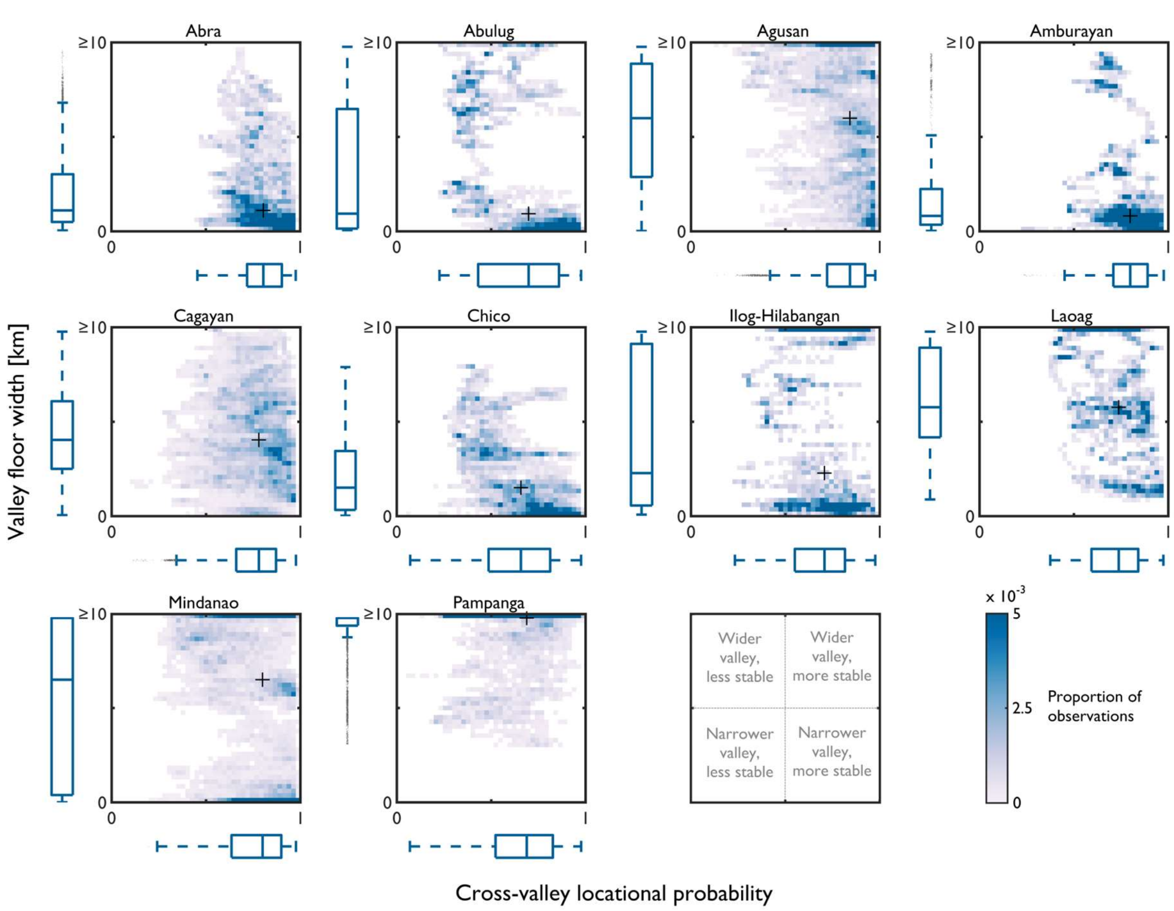

Supplementary Figure 4: Proportion of cross-valley locational probabilities displayed as a function of mapped valley floor width. Bin sizes are 0.025 for locational probability and 0.025 km for valley floor width. Marginal boxplots show the median and interquartile range; outliers are defined as values more than 1.5 times the interquartile range. Note that valley floor width is not smoothed (1 km moving average was only applied to Figure 3). Black marker with plus sign symbology denotes the median. Stable parts of the active channel are associated with a variety of valley floor widths. For one of the most stable channels (Abra), a substantial peak in frequency is located where locational probabilities are high (> 0.65) and valley floor widths are low (< 2 km). For a similarly stable channel (Agusan), multiple peaks in frequency are positioned where locational probabilities are high but distributed over the full range of valley floor widths (0 – ≥10 km). For the least stable channels (for example, Abulug and Chico) secondary peaks in frequency are associated with lower cross-valley locational probabilities at moderate to wide valley floors.

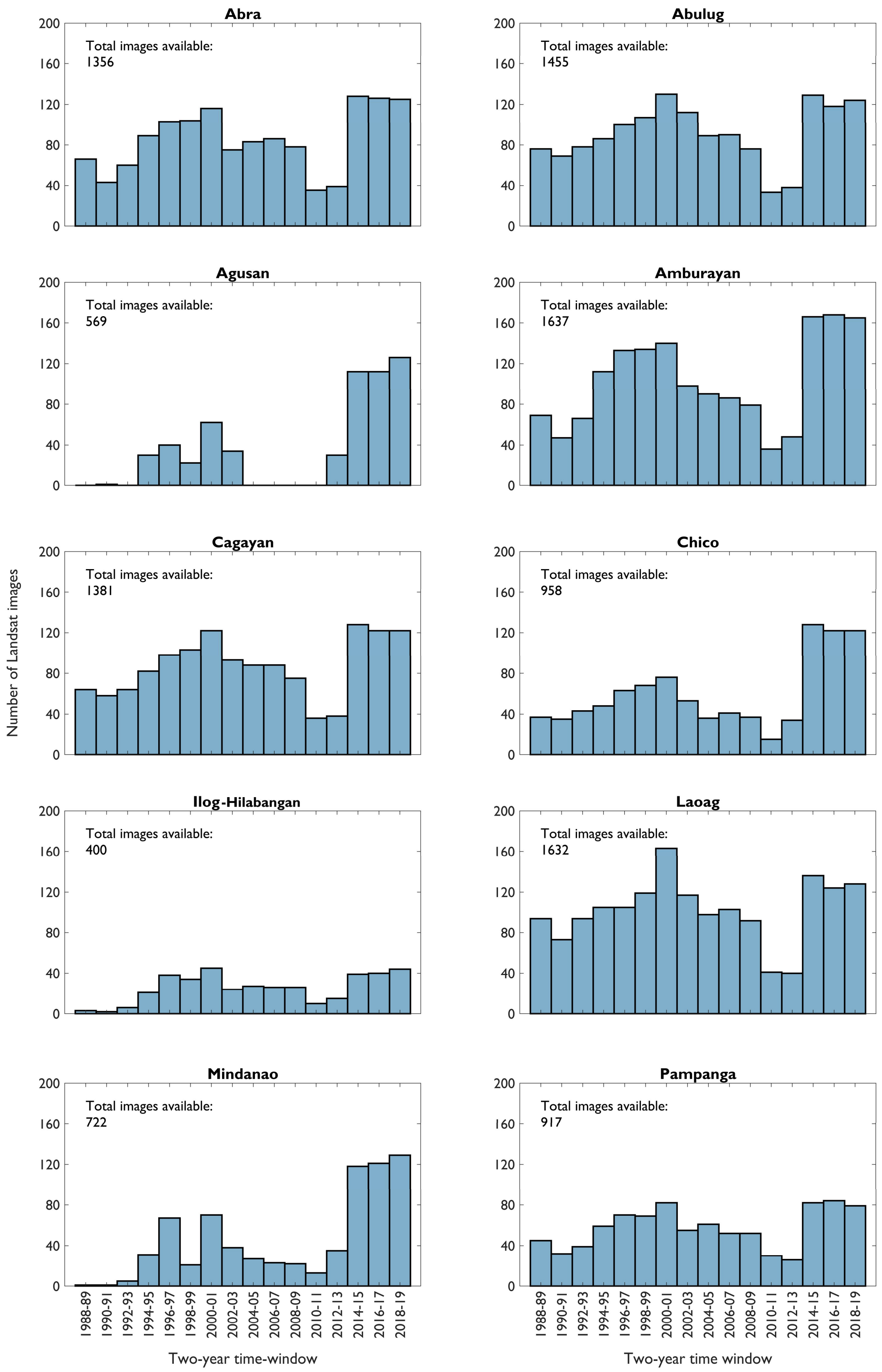

Supplementary Figure 5: Number of Landsat images covering the trunk channel region of interest for each two-year time window during the analysis period 1988-2019. The total number of Landsat images covering the trunk channel is displayed for each river.

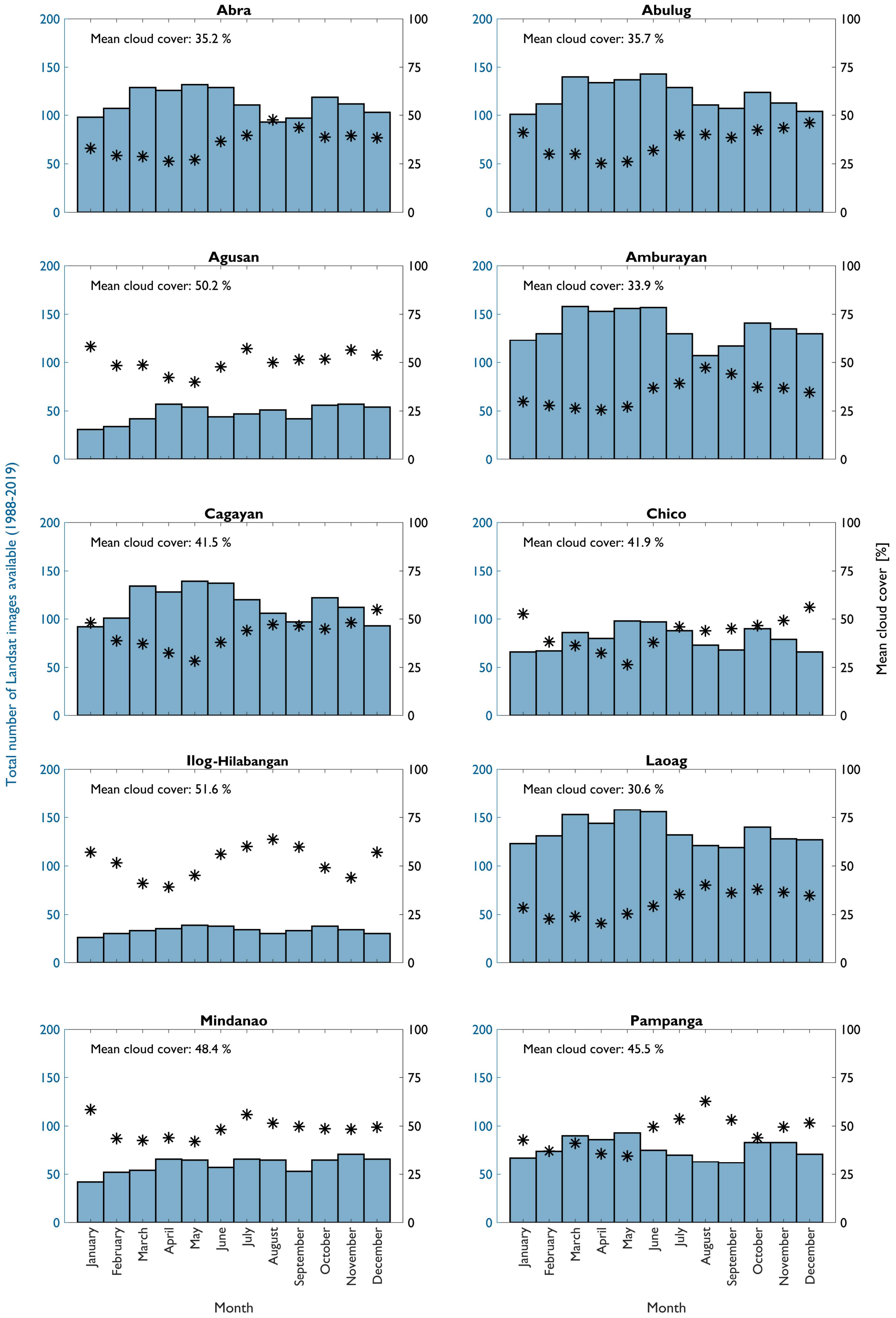

Supplementary Figure 6: Number of Landsat images covering the trunk channel region of interest each month during the analysis period 1988-2019. The image property "CLOUD_COVER_LAND" shows the percentage of clouds over land pixels in the Landsat scene. The image property provides an estimate of the average cloud cover each month and the average cloud cover over the entire analysis period for each river. Cloud-free pixels are retained in all months of the year, covering the full range of hydro-meteorological conditions.

Supplementary Table 1: Summary statistics of per-pixel locational probabilities (lp) over the full observable length of the 10 trunk channels. Trunk channels are sorted by the average per-pixel locational probability (listed from most stable to least stable).

Trunk channel	Total active channel area [km^2]	Average per-pixel locational probability [μ]	Proportion of active channel area where lp > 0.25	Proportion of active channel area where lp > 0.50	Proportion of active channel area where lp > 0.75	Proportion of active channel area where lp = 1 *
**Abra**	120.29	0.75	0.88	0.76	0.62	0.47
**Amburayan**	28.46	0.74	0.86	0.76	0.56	0.44
**Agusan**	36.15	0.74	0.90	0.72	0.57	0.51
**Cagayan**	233.03	0.71	0.83	0.71	0.59	0.44
**Laoag**	34.54	0.70	0.82	0.67	0.59	0.39
**Mindanao**	44.89	0.64	0.76	0.56	0.44	0.36
**Ilog-Hilabangan**	7.05	0.60	0.78	0.60	0.38	0.23
**Pampanga**	32.70	0.56	0.69	0.51	0.39	0.28
**Chico**	83.83	0.53	0.75	0.50	0.30	0.17
**Abulug**	55.07	0.51	0.68	0.43	0.26	0.19

* equivalent to 1 – λ in Figure 2a

Supplementary Table 2: Summary statistics of the local controls on geomorphic river mobility, assessed over constriction and expansion valley settings. Note that valley floor width is the mapped product (1 km moving average was only applied to Figure 3).

Valley setting	Re-scaled active channel width [-]		Re-scaled valley floor width [-]		Confinement ratio [-]		Cross-valley locational probability [-]	
	Average	Standard deviation	Average	Standard deviation	Average	Standard deviation	Average	Standard deviation
**Abra**	1.26	0.63	2.51	1.46	0.60	0.24	0.77	0.10
**Abulug**	3.27	1.69	9.79	8.58	0.52	0.28	0.52	0.21
**Chico**	2.54	1.40	8.73	6.04	0.43	0.27	0.57	0.16
**Cagayan**	1.64	1.21	8.58	4.01	0.25	0.20	0.78	0.14

Supplementary Table 3: Spearman correlation coefficients between local controls on geomorphic river mobility, assessed over constriction and expansion valley settings. Note that valley floor width is the mapped product (1 km moving average was only applied to Figure 3).

Valley setting	ACW – CVLP		VFW – CVLP		CR – CVLP		ACW – VFW		Observations
	Correlation coefficient	*p*-value	Correlation coefficient	*p*-value	Correlation coefficient	*p*-value	Correlation coefficient	*p*-value	
**Abra**	-0.50	<0.001	-0.09	<0.001	-0.14	<0.001	0.54	<0.001	3601
**Abulug**	-0.70	<0.001	-0.69	<0.001	0.46	<0.001	0.80	<0.001	2241
**Chico**	-0.80	<0.001	-0.35	<0.001	-0.05	0.0032	0.61	<0.001	3201
**Cagayan**	-0.37	<0.001	-0.01	0.5834	-0.22	<0.001	0.04	0.0872	2161

* ACW = active channel width; VFW = valley floor width; CR = confinement ratio; CVLP = cross-valley locational probability.

Supplementary Table 4: Summary statistics of the local controls on geomorphic river mobility, assessed over the full observable length of 10 trunk channels. Note that valley floor width is the mapped product (1 km moving average was only applied to Figure 3).

Trunk channel	Active channel width [km]		Valley floor width [km]		Confinement ratio [-]		Cross-valley locational probability [-]	
	Average	Standard deviation	Average	Standard deviation	Average	Standard deviation	Average	Standard deviation
**Abra**	0.92	0.74	2.11	2.15	0.62	0.29	0.82	0.12
**Abulug**	0.62	0.53	3.08	3.53	0.58	0.38	0.67	0.23
**Agusan**	0.28	0.24	5.96	3.29	0.10	0.15	0.82	0.16
**Amburayan**	0.79	0.59	1.99	2.63	0.72	0.30	0.81	0.12
**Cagayan**	0.71	0.52	4.46	2.42	0.22	0.19	0.77	0.15
**Chico**	0.64	0.52	2.25	2.12	0.46	0.28	0.67	0.19
**Ilog-Hilabangan**	0.20	0.14	4.35	4.03	0.16	0.18	0.70	0.17
**Laoag**	0.78	0.39	6.14	2.95	0.17	0.15	0.73	0.15
**Mindanao**	0.21	0.26	5.64	4.13	0.26	0.38	0.77	0.18
**Pampanga**	0.29	0.23	9.20	1.68	0.03	0.04	0.68	0.20

Supplementary Table 5: Spearman correlation coefficients between local controls on geomorphic river mobility, assessed over the full observable length of 10 trunk channels. Note that valley floor width is the mapped product (1 km moving average was only applied to Figure 3).

Trunk channel	ACW – CVLP		VFW – CVLP		CR – CVLP		ACW – VFW		Observations
	Correlation coefficient	*p*-value	Correlation coefficient	*p*-value	Correlation coefficient	*p*-value	Correlation coefficient	*p*-value	
**Abra**	-0.51	<0.001	-0.43	<0.001	0.16	<0.001	0.85	<0.001	11693
**Abulug**	-0.77	<0.001	-0.66	<0.001	0.57	<0.001	0.81	<0.001	8313
**Agusan**	-0.19	<0.001	-0.23	<0.001	0.08	<0.001	0.04	0.1986	11236
**Amburayan**	-0.60	<0.001	-0.53	<0.001	0.39	<0.001	0.92	<0.001	3149
**Cagayan**	-0.30	<0.001	-0.10	<0.001	-0.18	<0.001	0.41	<0.001	28591
**Chico**	-0.81	<0.001	-0.67	<0.001	0.23	<0.001	0.85	<0.001	11924
**Ilog-Hilabangan**	-0.56	<0.001	-0.06	0.0010	-0.11	0.6184	0.38	<0.001	2613
**Laoag**	-0.27	<0.001	-0.47	<0.001	0.19	<0.001	0.09	<0.001	3859
**Mindanao**	-0.41	<0.001	-0.15	<0.001	0.01	0.1408	0.46	<0.001	18654
**Pampanga**	-0.67	<0.001	0.22	<0.001	-0.68	<0.001	-0.19	<0.001	10243

* ACW = active channel width; VFW = valley floor width; CR = confinement ratio; CVLP = cross-valley locational probability.

Supplementary Table 6: Topographic properties of the 10 Philippine catchments. Topographic data were from a nationwide IfSAR-derived DEM acquired in 2013 [1]. The DEM was resampled to 10 m spatial resolution and catchments were processed using TopoToolbox V2 [2].

Catchment	Catchment area [km²]	Catchment relief [m]	Catchment median elevation [m]	Average catchment slope [°]	Average channel slope [m/m]
**Abra**	4893.42	2578.32	669.42	22.35	0.05
**Abulug**	2815.71	2356.77	433.96	19.38	0.04
**Agusan**	11528.56	2665.46	135.34	13.79	0.02
**Amburayan**	1273.68	2716.44	957.25	28.10	0.09
**Cagayan**	27684.07	2871.04	337.11	16.62	0.03
**Chico**	4962.34	2680.15	694.47	20.29	0.05
**Ilog-Hilabangan**	1985.67	1469.58	239.64	12.66	0.02
**Laoag**	1261.60	2355.70	231.04	17.42	0.04
**Mindanao**	18512.80	2945.90	353.14	12.09	0.03
**Pampanga**	7842.33	1896.83	75.40	9.13	0.01

Supplementary Table 7: Climate type, rainfall, geology and land cover properties of the 10 catchments. Climate type data were from [3]. Rainfall data were from the end-of-the-day adjusted version of the APHRODITE data set (V1901; [4]). Geology data were from Mines and Geosciences Bureau (MGB) 2010 geology and mineral resources data set. Land cover data were from the National Mapping and Resource Information Authority (NAMRIA) 2010 land cover data set (www.namria.gov.ph).

Catchment	Mean annual rainfall [mm]	Range in mean annual rainfall [mm]	Primary climate type *	Proportion of catchment area [%]	Primary lithology	Proportion of catchment area [%]	Primary land cover	Proportion of catchment area [%]
Abra	2379.2	359.9	I	100.0	Undifferentiated metavolcanics	41.7	Wooded Grassland	31.6
Abulug	1953.6	986.9	III	73.4	Undifferentiated metavolcanics	40.6	Shrubs	26.4
Agusan	2407.7	1577.9	IV	91.6	Marl, reworked tuff, pyroclastic	37.7	Open Forest	29.9
Amburayan	2449.2	832.2	I	100.0	Sandstone, shales, reef limestone	60.2	Wooded Grassland	33.9
Cagayan	1880.6	1572.2	III	85.8	Sandstone, shales, reef limestone	23.6	Annual Crop	31.3
Chico	2020.4	976.8	III	66.5	Sandstone, shales, reef limestone	24.3	Closed Forest	21.4
Ilog-Hilabangan	2326.7	516.0	III	100.0	Pliocene-Pleistocene limestone	33.3	Annual Crop	44.5
Laoag	2199.6	466.7	I	100.0	Recent deposits	33.1	Wooded Grassland	28.6
Mindanao	1951.9	1166.2	III	83.9	Recent deposits	25.7	Annual Crop	42.1
Pampanga	2089.9	1119.8	I	66.0	Recent deposits	56.5	Annual Crop	51.9

* Modified Coronas climate classification: Type I = distinct dry period from November to April and a wet season from May to October; Type II = rainfall evenly distributed throughout year, but with pronounced rainy season from November to January; Type III = seasons not very pronounced, but a relatively dry period prevails from November to April; and Type IV = rainfall is evenly distributed throughout the year.

Supplementary Table 8: Temporal coverage and total number of time-windows of binary active channel imagery with sufficient quality for geomorphic river mobility analysis.

Trunk channel	Time-windows included in channel mobility analysis																Number of time-windows included (x)
	1988-89	1990-91	1992-93	1994-95	1996-97	1998-99	2000-01	2002-03	2004-05	2006-07	2008-09	2010-11	2012-13	2014-15	2016-17	2018-19	
**Abra**																	14
**Abulug**																	16
**Agusan**																	6
**Amburayan**																	16
**Cagayan**																	13
**Chico**																	15
**Ilog-Hilabangan**																	13
**Laoag**																	14
**Mindanao**																	8
**Pampanga**																	14

Supplementary References

1. Grafil, L. B. & Castro, O. T. Acquisition of IfSAR for the Production of Nationwide DEM and ORI for the Philippines under the Unified Mapping Project. *Infomapper* **21**, 12–13, 40–43 (2014).
2. Schwanghart, W. & Scherler, D. Short Communication: TopoToolbox 2 - MATLAB-based software for topographic analysis and modeling in Earth surface sciences. *Earth Surface Dynamics* **2**, 1–7 (2014).
3. Tolentino, P. L. M. *et al.* Projected impact of climate change on hydrological regimes in the Philippines. *PLoS ONE* **11**, e0163941 (2016).
4. Yatagai, A. *et al.* Aphrodite constructing a long-term daily gridded precipitation dataset for Asia based on a dense network of rain gauges. *Bulletin of the American Meteorological Society* **93**, 1401–1415 (2012).
